# Potential of Modification of Techno-Functional Properties and Structural Characteristics of Citrus, Apple, Oat, and Pea Dietary Fiber by High-Intensity Ultrasound

**DOI:** 10.3390/foods12193663

**Published:** 2023-10-04

**Authors:** Ann-Marie Kalla-Bertholdt, Anne Kathrin Baier, Cornelia Rauh

**Affiliations:** Department of Food Biotechnology and Food Process Engineering, Technische Universität Berlin, Koenigin-Luise-Str. 22, 14195 Berlin, Germany

**Keywords:** high-intensity ultrasound, soluble dietary fiber, insoluble dietary fiber, fiber functionalization, water binding capacity, rheological properties, microstructure

## Abstract

Plant fibers are rich in dietary fiber and micronutrients but often exhibit poor functionality. Ultrasonication can affect the particle size of plant fiber, thereby influencing other techno-functional properties. Therefore, this study aimed to investigate the effects of high-intensity ultrasound on citrus, apple, oat, and pea fiber. Initially, solutions containing 1 wt% of plant fiber were homogenized using ultrasonication (amplitude 116 µm, t = 150 s, energy density = 225 kJ/L, $\bar{P}\;$= 325 W). Due to cavitation effects induced by ultrasound, differences in particle size and a shift in the ratio of insoluble and alcohol-insoluble fractions for dietary fiber were observed. Additionally, viscosities for citrus and apple fiber increased from 1.4 Pa·s to 84.4 Pa·s and from 1.34 Pa·s to 31.7 Pa·s, respectively, at shear rates of 100 $\frac{1}{s}$. This was attributed to observed differences in the microstructure. Freeze-dried samples of purified citrus and apple fiber revealed thin and nearly transparent layers, possibly contributing to enhanced water binding capacity and, therefore, increased viscosity. Water binding capacity for citrus fiber increased from 18.2 g/g to 41.8 g/g, and a 40% increase was observed for apple fiber. Finally, ultrasound demonstrated itself be an effective technology for modifying the techno-functional properties of plant fiber, such as water binding capacity.

## 1. Introduction

The processing of fruits, vegetables, and cereals generates large quantities of agricultural waste material yet contains highly nutritious components. Particularly regarding the increasing demand for food, it is of major interest to transform these by-products into valuable ingredients. Especially dietary fibers (DF), as the predominant nutrient in these by-products, are important for human health. Recent studies have shown that the fermentation of DF in the human intestine produces short-chain fatty acids, having a positive impact on common chronic conditions such as type 2 diabetes or coronary heart diseases [1]. However, fermentability is not the only characteristic of DF that is beneficial for human health. A common classification of DF is based on both their fermentability and water solubility. Water-soluble DF are also well fermented and include gums and pectic substances. Whereas water-insoluble DF are less fermented and include cellulose, hemicellulose, and lignin [2]. Pectin is a heteropolysaccharide composed of as many as eighteen distinct monosaccharides, which can form three major domains, namely homogalacturonan (HG), rhamnogalacturonan I (RG-I), and rhamnogalacturonan II (RG-II). The major chain of HG and RG-II is composed of α-D-galacturonic acid residues (GalA) as the predominantly basic unit, which is linked via α-1,4 glycosidic bonds. For RG-I, the backbone is comprised of rhamnose-GalA dimers. For HG, regions where only unbranched chains of α-1,4 GalA are found are characteristic. HG is the most abundant domain of pectins. The linear polymer chain can be modified due to methyl- or acetylesterification at the C-6 and O-2/O-3 positions, with complex side chains, resulting in RG-II. RG-I, instead, is made of alternating GalA and rhamnose residues. Rhamnose can be branched by arabinose- or galactose-containing side chains with varying complexity [3,4]. Cellulose is a polysaccharide, generally comprised of 10,000 to 15,000 glucose units per chain. Via β-D-1,4 glycosidic bonds between the glucose molecules, a linear structure without any branching is formed. Due to hydrogen bonding and Van der Waals forces between cellulose chains, rigid, nonflexible, and crystalline microfibrils are formed. Then, these microfibrils assemble into macroscopic fibers with diameters of 5–20 µm. This structure is responsible for its insoluble, dense, and enzyme-resistant behavior [5,6]. Hemicelluloses have a backbone chain composed of different β-D-1,4 glycosidic linked monosaccharides alone or in combination, such as glucose, galactose, arabinose, or xylose. Xylans are the most abundant type of hemicelluloses, having a xylopyranosyl backbone with sidechains attached at the C-2 or C-3 position. Due to attached side chains, such as arabinofuranose or glucuronic acids, a complex branched structure is formed. Hemicelluloses are comprised of 50 up to 200 monomeric units and are also, similar to cellulose, insoluble in water but soluble in alkaline solutions [5,7]. In general, insoluble substances are beneficial due to their enhanced swelling capacity. Swelling of DF results in increased fecal bulk and increased intestinal transit time, simultaneously reducing the efficacy of toxic substances [5,8]. Besides their positive health effects, DF have distinct techno-functional properties that may influence the characteristics of a food product, such as viscosity, emulsifying, or water binding capacity [9,10,11]. The desired properties may vary within the field of application. For improving the properties of food products, such as stability or appearance, DF with distinct functionalities are required. If the sole purpose of enrichment is based on health aspects, DF should only have a minor impact on the product’s properties. High-intensity ultrasound as an innovative and green technology is applied to transform dietary fiber into value-added products with improved and product-specific techno-functional properties [12]. The underlying mechanism of ultrasound is called cavitation. The literature describes two different types of cavitation that happen during sonication. During stable cavitation, small vapour-filled bubbles are generated, traveling through the medium, causing rotational movement of the fluid and, therefore, homogenous mixing. On the other hand, transient cavitation is characterized by the rapid formation and collapse of large-sized vapour-filled bubbles within a short time, causing pressure maxima, hot spots, and enormous shear forces in the liquid sample, where particles and droplets are ruptured [13,14]. There is a lot of literature available on the influence of ultrasound on purified insoluble or soluble DF, such as pectin or cellulose [15,16,17,18]. Nevertheless, achieving a high degree of purification involves multiple processing steps and increased energy consumption. Hence, considering sustainability and clean-labeling reasons, there is significant interest in less isolated or purified compounds. Consequently, the application of high-intensity ultrasound on commercial plant DF is of special interest to modify its techno-functional characteristics. However, to this date, little is known about the effect of ultrasound on commercial plant fiber compounds containing both insoluble and soluble DF with varying degrees of purification. The interactions between these two types of DF, as well as their influence on techno-functionality, are not properly understood. Previous investigations by Kalla-Bertholdt et al. [19,20] have revealed several modified properties due to the sonication of fiber solutions, such as increased viscosity or altered bioaccessibility of lipids or proteins, which have not yet been thoroughly identified. Therefore, this study aimed to investigate the effect and the underlying mechanisms of ultrasound with constant power input on five different plant dietary fibers, namely apple, oat, pea, and two different citrus fibers.

## 2. Materials and Methods

### 2.1. Materials

Commercial dietary fiber compounds (citrus fiber Herbacel AQ Plus Citrus N; citrus fiber Herbacel Classic CF 02; apple fiber Herbacel AQ Plus Apple A 09; oat fiber Herbacel Classic Plus HF 04; and pea fiber Herbacel Classic Plus EF 01) were received from Herbafood Ingredients GmbH (Werder, Germany). All other chemicals used in this study were purchased from Sigma-Aldrich (St. Louis, MO, USA) or Merck KGaA (Darmstadt, Germany) unless stated otherwise.

### 2.2. Sample Preparation Using High-Intensity Ultrasound

Samples were prepared, each containing 1 wt% of plant dietary fiber in a 200 mL volume. Various dietary fibers, including apple, pea, oat, and two distinct citrus fibers with differing levels of purification, were utilized. Following the mixing of the dietary fiber with tap water, the pH of the solution was adjusted to 7 and stirred for 10 min. To homogenize the solution, high-intensity ultrasound (UIP 2000; BS2s18 (F), Hielscher Ultrasonics GmbH, Berlin, Germany) was employed under the following conditions: amplitude = 116 µm, t = 150 s, energy density = 225 kJ/L, and $\bar{P}$ = 325 W. Throughout the ultrasound treatment, the sample was placed in an ice bath and manually stirred. The temperature in the suspension was monitored using a manual thermometer (testo 104, Testo, Lenzkirch, Germany) and did not exceed 40 °C. Additionally, a control sample was prepared following the same procedure, but without undergoing sonication, to assess the impact of ultrasound treatment on dietary fiber.

### 2.3. Characterization of Plant Dietary Fiber

#### 2.3.1. Composition of Plant Dietary Fibers

Table 1 presents a comprehensive overview of the composition and total dietary fiber content (TDF) of the plant dietary fiber used in this study. This information was provided by the manufacturer. The soluble and insoluble dietary fiber content was quantified according to the AOAC procedure No. 991.43, employing the Total Dietary Fiber Assay Kit from Megazyme (Wicklow, Ireland) as detailed in Table 2. For reference, the abbreviations used for different fiber types are as follows: CF AQ—Citrus fiber AQ Plus Citrus N; CF Cl—Citrus fiber Herbacel Classic CF 02; AF—Apple fiber Herbacel AQ Plus Apple A 09; OF—Oat fiber Herbacel Classic Plus HF 04; and PF—Pea fiber Herbacel Classic Plus EF 01.

#### 2.3.2. Determination of Pectin Content (GalA)

As an indicator of pectin content, the content of galacturonic acid (GalA) was determined. Initially, 100 mg of each plant fiber was suspended in 45 mL of a 0.5% suspension of Chelaplex III. The pH value was adjusted to 11.8 and the suspension was incubated at room temperature for 1 h. Afterwards, the pH value was decreased to 4.5, and 2 mL of a 1 v% enzyme solution was added, containing Vegazym M (Erbslöh Gelsenheim GmbH, Gelsenheim, Germany) and Celluclast (3:1; Novozymes A/S, Bagsvaerd, Denmark), followed by another incubation at room temperature for 15 h. Finally, the liquid fraction was recovered by filtration and diluted appropriately for the determination of galacturonic acid, following the method of Blumenkrantz and Asboe-Hansen [21] with slight modifications. Therefore, the solubilized sample was acidified with a sulfuric acid tetraborate solution (6:1, *v*:*v*) and boiled for 10 min. Before adding 50 µL of m-hydroxybiphenyl solution, the samples were cooled down to room temperature in a water-ice bath. After 20 min of incubation, the absorbance was measured at 520 nm. 0.5% NaOH was used as a blank, and galacturonic acid monohydrate was used for the calibration curve.

#### 2.3.3. Determination of Insoluble, Alcohol-Insoluble, and Soluble Fractions in Dietary Fiber Suspensions before and after US-Treatment

The characterization of dietary fiber solutions regarding insoluble fractions (IF), alcohol-insoluble fractions (AIF), and soluble fractions (SF) was conducted by adapting the method of Morales-Medina et al. [22]. IF represent water-insoluble components, whereas AIF are soluble in water but not in ethanol, and SF are soluble in both water and ethanol. Briefly, plant fiber and water were mixed thoroughly, and for treated samples, ultrasound was applied as well. The fiber contents in the solutions needed to be adjusted because of the high soluble dietary fiber contents or water binding capacity of some of the used plant fibers. For the exact amount of each fiber added, see Table 3.

As a first step after mixing or sonicating the suspensions, the total volume was filtered through filtration bags (FibreBag S, 10-ß142, Gerhardt GmbH & Co Kg, Königswinter, Germany). The insoluble residue was washed twice with 10 mL distilled water (preheated to 60 °C), and washings were collected in the filtrate. After that, the retentate was washed twice with each, 15 mL of ethanol (95 v%) and acetone. The washings were discarded, and the filtration bag containing the IF was dried overnight at 105 °C and weighed accurately. For AIF, 50 mL of the filtrate from step one was mixed with 200 mL of preheated ethanol (60 °C, 95 v%) and allowed to stay for 1 h at room temperature. Again, the precipitate was then filtered through a filtration bag and washed twice with each: 15 mL of ethanol (78 v%), 15 mL of ethanol (95 v%), and 15 mL of acetone. This bag, containing AIF, was also dried overnight at 105 °C and weighed accurately. The SM was calculated using the following equation:

(1) $$m_{DM}=m_{IF}+m_{AIF}+m_{SF}$$

where $m_{DM}$ is the dry weight of the initial amount of fiber added, $m_{IF}$ is the mass of the insoluble fraction and $m_{AIF}$ represents the mass of the alcohol-insoluble fraction. The IF, AIF, and SF are represented as percentages of the total dried matter and experiments were conducted at least in duplicate. In this work, IF are assumed as an approximation for insoluble dietary fiber and AIF for soluble dietary fiber.

#### 2.3.4. Water Binding Capacity

The water binding capacity (WBC) of the examined plant fibers was measured before and after ultrasound treatment. Therefore, a 1 wt% fiber solution was prepared and sonicated according to the sample preparation parameters (see Section 2.2). The untreated samples were stirred for 10 min only. Afterwards, 30 mL of the solution was centrifuged for 15 min at 3000× *g* (centrifuge 5804 R, Eppendorf AG, Wesseling-Berzdorf, Germany). The supernatants were discarded, and the tubes were placed upside down for a further 10 min, draining the unbound water. Then, 1 g of the remaining pellet was weighed accurately (±0.001 g) in dried glass cups, followed by drying in a drying oven (Heraeus Instruments, UT 6060, Hanau, Germany) at 105 °C for 24 h. Finally, the glass cups containing dried pellets were weighed, and WBC was calculated as follows:

(2) $$WBC\;\left[\frac{g_{water}}{g_{fiber}}\right]=\frac{m_{2}-m_{0}-m_{1}}{m_{1}}$$

where $m_{0}$ is the weight of the glass cup, $m_{1}$ is the initial weight of the pellet (1 ± 0.001 g) and $m_{2}$ is the final weight of the drained pellet and glass cup.

#### 2.3.5. Swelling Capacity

The swelling capacity of plant fiber samples was examined in duplicate following the method published by Zhang et al. [23], with slight modifications. Initially, 0.2 ± 0.01 g of sample was gradually mixed with 10 mL of distilled water in a 15 mL measuring cylinder and left to stand for 24 h at 6 °C. The volume fractions occupied by the dry sample before ($V_{1})$ and after 24 h of incubation ($V_{2})$ were recorded. The SC was expressed as a volumetric change of the plant fiber sample after water swelling as follows:

(3) $$SC\left[\frac{ml}{g}\right]=\frac{V_{2}-V_{1}}{m_{0}}$$

where $m_{0}$ is expressed as the dry mass of the initial weighed sample. The untreated and ultrasound-treated plant fiber samples were freeze-dried and milled in a mill after sonication.

#### 2.3.6. Determination of Particle Size Distribution

The investigation of the particle size distribution of the untreated and ultrasound-treated fiber suspensions was conducted following the method previously reported by Kalla-Bertholdt et al. [20]. All suspensions were measured at least in triplicate.

#### 2.3.7. Rheological Characterization

To measure the rheological characteristics of the suspensions containing 1% fiber (UT/T), the methods of Huang et al. [24] and Kalla-Bertholdt et al. [20] were applied.

#### 2.3.8. Field Emission Scanning Electron Microscopy (FE-SEM)

For microstructural analysis, samples were analyzed following the method previously applied by Kalla-Bertholdt et al. [20].

#### 2.3.9. Statistical Analysis

All experiments were conducted in duplicate or as otherwise specified. Standard deviations and averages were calculated based on these measurements. To assess significant differences between untreated and treated samples, a student’s *t*-test was employed. Statistical significance was considered at *p* < 0.05. Microsoft Excel (Version 2307) was utilized for statistical analysis.

## 3. Results and Discussion

**Figure 1:**
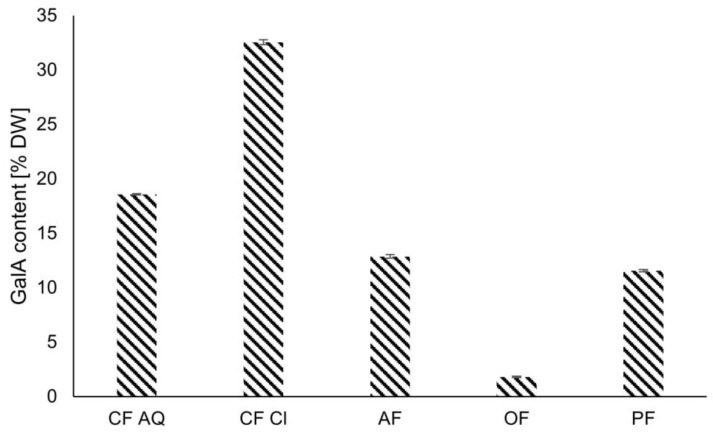
Galacturonic acid content [% DW] as an indicator for pectin in the different plant fiber samples. CF AQ—Citrus fiber AQ plus; CF Cl—Citrus fiber classic; AF—Apple fiber; OF—Oat fiber and PF—Pea fiber.

The galacturonic acid content (GalA) of the used plant dietary fiber was investigated as an indicator for pectin, which is the most abundant soluble dietary fiber (SDF) in fruits (Figure 1). GalA is the basic unit of pectin molecules and is linked via α-1-4 glycosidic bonds. In general, pectin is a heterogenic and versatile group of heteropolysaccharides. Its structure is based on origin, extraction method, and enzymatic modification. Homogalacturonans are regions in pectin molecules where only GalA molecules are found. In addition, other structural regions or complex side chains are found: rhamnogalacturonan I or II, xylogalacturonan, and apiogalacturonan [25]. Pectin can be classified into three different categories: free pectin, pectic acid, and protopectin. Free pectin naturally occurs in fruit and soft places only on the periphery of the cell wall. It is a fully methylated ester of pectic acid and is the only pectin type that is water-soluble. In addition, it has the highest gelling capacity. Pectic acid, however, is the product of the hydrolysis of any pectic substance and is insoluble in water. Finally, protopectin is a methyl ester of pectic acid and occurs in the inner part of the cell wall, closest to the cellulose layer directly located under the free pectin layer. Protopectin is considered a cellulose compound, even if there is no clear distinction between locations in the cell wall of free pectin and protopectin. Protopectin is formed due to carboxyl links between pectin and cellulose, where methoxyl groups are replaced by cellulose groups. Heat is recognized as a responsible factor for the hydrolysis of “free pectin” from protopectin. This newly generated free pectin is not only less methylated than the “original” free pectin but also soluble in water [26]. The results of the GalA content determination of the used plant fibers are shown in Figure 1.

It was expected that CF Cl would exhibit high amounts of GalA because of the maximum amount of SDF detected among the plant DF used in this study. Additionally, the lowest amounts of GalA were anticipated for OF, as the most abundant SDF in oats is β-glucan, not pectin [27]. β-glucan is composed of D-glucose monomers linked via β-glycosidic bonds. Primarily, these d-glucopyranosyl units are connected either by isolated β-(1-3) linkages or a set of β-(1-4) linkages. The β-(1-3) linkages form bends, resulting in a worm-like coil structure and causing water solubility [28]. For PF, a low to medium amount of pectic substances was assumed due to the low amount of SDF content detected and the constituents of the used pea DF material. Dietary fibers obtained from peas are classified into inner and outer fibers, referring to the cell wall material of the cotyledon and hull, respectively. The inner fraction of the cotyledon consists of cell wall polysaccharides with varying degrees of solubility, whereas the outer part of the hull is dominated by insoluble polysaccharides and some pectin [29]. Additionally, pea DF, as a by-product generated during starch or protein extraction, was assumed to be only slightly different from the GalA contents of raw, dry pea DF. In study, reviewing the compositions of different raw, dry legumes and pulses, this assumption was verified. The GalA content of yellow pea dietary fiber appeared to be between 15.6 and 18.4%, which is slightly higher than the values obtained in this study of 11.57% [30]. For AF, CF AQ, and CF Cl, results were not as expected. As outlined in a later section, after ultrasound treatment of solutions containing AF and CF AQ, there was an enormous increase in viscosity Therefore, it was assumed that ultrasound releases pectin out of the protopectin matrix, resulting in the gelling of the solution. This, in turn, led to the assumption that the SDF content is underestimated by the amount of protopectin, which is insoluble in its native state and possibly becomes soluble after ultrasound treatment. To elaborate on this hypothesis in more detail, the methods for determining SDF and GalA, respectively, need to be discussed. The biggest methodological difference in IDF/SDF determination of AOAC procedure No. 991.43 and the applied GalA content measurement is the utilization of different enzymes. The AOAC method employs enzymes similar to those existing in human digestion (namely pepsin, α-amylase, and amyloglucosidase), leading to the degradation of molecules accessible for these types of enzymes. As is known, enzymes in the human body are not able to hydrolyze DF in general, especially cellulose or pectin. However, for the applied GalA method, Vegazym M and Celluclast were used, containing pectinases and cellulases. In fact, this method breaks down the complete cell wall material into monomers and solubilizes them. This means that insoluble protopectin, which is bound to cellulose, will be released, solubilized, and detected in the GalA method but not in the SDF measurement used by AOAC. Therefore, it was hypothesized that AF and CF AQ would have the highest amounts of GalA because ultrasound treatment will release cellulose-bound pectin (protopectin) out of remaining intact cell wall fragments, resulting in the gelling of the liberated protopectin molecules. As seen in Figure 1, CF CL has the highest amounts of GalA with 32.57% DW, followed by 18.59% DW for CF AQ and 12.88% DW for AF. On the one hand, these values are only slightly higher than SDF content, and on the other hand, both CF AQ and AF do not contain the highest amounts of GalA compared to CF Cl. The overall differences between the GalA contents of these three fiber compounds might be found in the processing history. AF as well as CF AQ are the residual products of pectin extraction, resulting in lower amounts of pectic substances. CF Cl, instead, is a more unrefined material, containing higher amounts of remaining pectic components. Overall, the GalA content in the fiber compounds decreases in the following order: CF Cl ≥ CF AQ ≥ AF ≥ PF ≥ OF.

**Figure 2:**
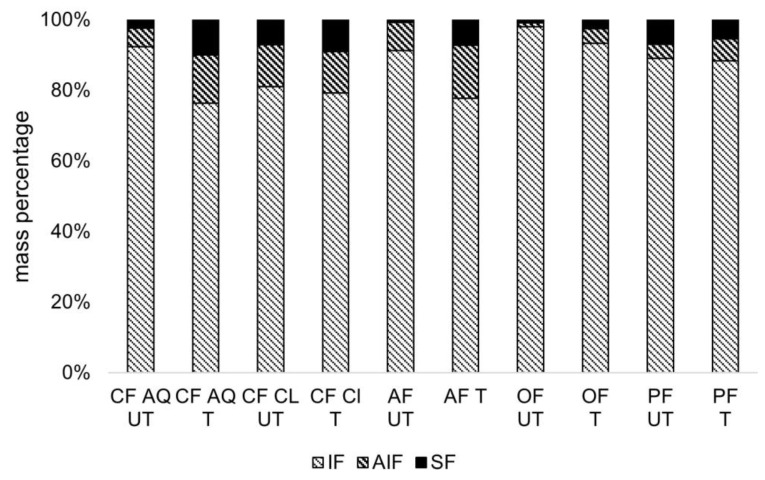
Mass composition in relation to insoluble, alcohol-insoluble, and soluble fractions of the untreated (UT) and ultrasound-treated (T) fiber suspensions (CF AQ—Citrus fiber AQ Plus; CF Cl—Citrus fiber classic; AF—Apple fiber; OF—Oat fiber; PF—Pea fiber).

To gain a deeper understanding of the effect of ultrasound treatment on dietary fiber, the insoluble fractions (IF), alcohol-insoluble fractions (AIF), and soluble fractions (SF) were determined (Figure 2). The IF represents mostly insoluble dietary fiber (IDF), including cellulose, hemicellulose, or lignin, and negligible amounts of ashes and protein. The AIF represent an approximation of mostly soluble dietary fiber (SDF), including pectic-like substances and branched polysaccharides, such as sections of hemicellulose with a degree of polymerization greater than 12. SF refer to substances soluble in both water and ethanol [22,31]. It is known that porosity and particle size can be determining factors for the interaction of substances with water due to capillary attraction or exposure of hydrophilic groups [32]. As a result, solubility can be modified by mechanical treatment [33]. Many studies revealed a shift from IDF to increased SDF content after sonication [33,34,35]. This phenomenon is attributed to cavitation-induced loosening of the fiber tissue and, finally, breakage of polymeric chains without changing the primary structure [36,37]. In a study investigating the influence of ultrasonication on the physicochemical and structural properties of SDF from corn bran, the monosaccharide composition of extracted SDF was analyzed. It was concluded that connections between cellulose and hemicellulose or lignin were destroyed, and especially hemicelluloses, having a lower degree of polymerization, are further degraded and dissolved [38]. As seen in Figure 2, the IF, AIF, and SF for all tested fiber compounds before and after ultrasound treatment are illustrated. For CF Cl, OF, and PF, only minor changes are detectable, which are not significant. For OF, the initial particle size might be crucial). In a study evaluating the effect of ultrasound on citrus pectin, degradation below a critical size is limited, and therefore, no effect can be seen [39]. For CF Cl and PF, representing less purified fiber compounds, the presence of remaining carbohydrates and proteins (Table 1) could lead to varying results. Concerning the results of CF, AQ, and AF the amount of AIF, representing an approximation for SDF, is significantly increased after sonication (Figure 2). Morales-Medina et al. [22] demonstrated an increase in AIF in pea hulls, modified by microfluidization. This was referred to as the defibrillation of macrofibrils into microfibrils of cell wall polysaccharides. The energy of the microfluidization process was high enough to (i) partially defibrillate microfibrils, thus releasing insoluble fibrils, (ii) break the interactions between hemicellulose-cellulose, hemicellulose-pectin, and pectin-cellulose and (iii) break the glycosidic bonds within polysaccharides such as cellulose, hemicellulose, and pectin. As a result, the decomposition of hemicellulose and pectin might lead to an increase in AIF. Potentially, some of these assumptions are applicable for ultrasound treatment, too. For CF AQ and AF, which are highly purified by-products of pectin extraction, the harsh processing and extraction conditions might have led to a weakening of the fiber structure. This, in turn, could have influenced the effect of sonication on the degree of decomposition of hemicellulose and pectin, resulting in an increase in AIF.

**Figure 3:**
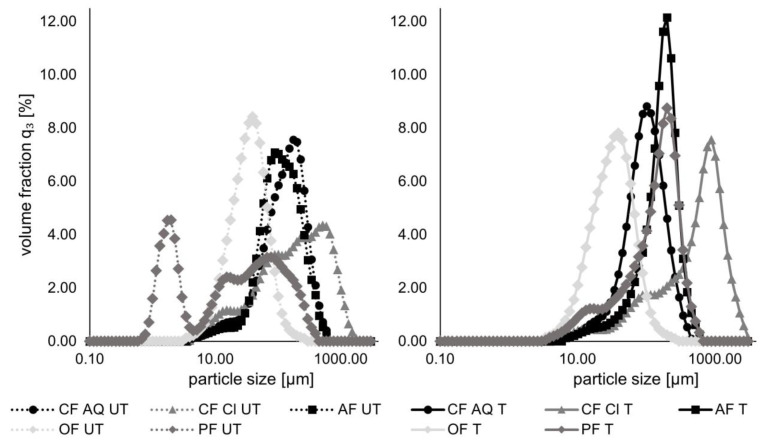
Particle size distribution of untreated (**left**) und ultrasound-treated (**right**) fiber suspensions (CF AQ—Citrus fiber AQ Plus; CF Cl—Citrus fiber classic; AF—Apple fiber; OF—Oat fiber; PF—Pea fiber).

In Figure 3, the particle size distributions of all untreated and ultrasound-treated fiber suspensions are illustrated. Several studies investigating the effect of high-intensity ultrasound on particle size distributions of different plant fibers revealed particle size reductions among different applied intensities [18,40]. The findings of this study are predominantly opposite. For untreated samples of CF AQ, AF, and OF, a broad monomodal distribution was found. For CF Cl and PF, a bimodal distribution in a wide range was detected. This could be due to the presence of residual substances of various sizes, such as starch for PF or other components in the CF Cl sample, which is the least refined among all the investigated fiber compounds. After ultrasound treatment, the curves became narrower, and a monomodal distribution with increased volume fractions was observed. This finding is in accordance with a study investigating the effect of ultrasound on rice starch. They concluded the shift from bimodal to a narrow unimodal particle size distribution might be attributed to more homogenous particles created by sonication [41]. In another study, preparing physically modified oat starch with different ultrasound treatments, similar results were found. They suggested that the enhanced homogeneity of particles is due to the disruption of larger starch agglomerates rather than the disintegration of small individual granules [42]. For the investigated fiber particles in the current study, these assumptions might be transferable. As seen in the FE-SEM images for pea fiber in a later section), the initially present single particle composites are broken down into smaller pieces of similar size. For AF and CF AQ, the inhomogeneous structures are homogenized as well. Oat fiber particles are only slightly different in size. Besides a slight reduction, the roughening of the surface is more prominent. Only CF Cl is an exception to these findings. The microstructure revealed no single particles but rather loose fiber bundles. After ultrasound treatment, the fiber bundles exhibited finer structures, consisting of thin filaments. Sonication seems to enhance the entanglement of CF Cl filaments. The shift of the peak towards particle sizes around 1000 µm might be attributed to the detection of bigger filamentous aggregates rather than single fiber filaments. In turn, the phenomenon of smaller particles in FE-SEM images, but mostly increasing particle size measured by laser scattering (Figure 3), might be attributable to the measurement of aggregated particles rather than single pieces.

**Figure 4:**
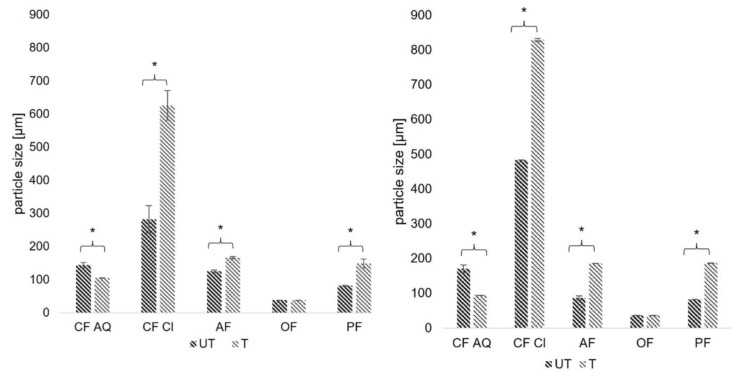
Mean particle size (**left**) and mode (most commonly occurring particle size; **right**) for all untreated and ultrasound-treated fiber samples [µm] (CF AQ—Citrus fiber AQ Plus; CF Cl—Citrus fiber classic; AF—Apple fiber; OF—Oat fiber; PF—Pea fiber). Columns marked with “*” indicate significant differences between UT and T samples (*p* < 0.05).

In Figure 4, the mean particle diameter (left) and the mode, as well as the most occurring particle size (right), are shown. The basis for SLS measurements is the assumption of a spherical particle with an equivalent diameter and a constant volume. This simplification only approximates fiber particles to a certain extent, which in turn can lead to minor variations compared to ideal spherical particles. Nevertheless, the influence of cavitation-induced shear forces might not only be dependent on ultrasound power and treatment time but also on the nature of plant fiber samples, especially remaining substances other than dietary fiber, and initial particle size. In a study investigating the effect of ultrasound on okara tofu analogues, okara fibers were treated with different sonication intensities ranging from 500 to 800 W for 20 min [43]. They received for untreated fiber samples particle sizes around 140 µm and a broad distribution between 5 and 635 µm. After ultrasound treatment, the distribution was narrower and the volume fraction increased, like in the results of this study. Interestingly, exceeding power levels above 600 W did not result in further particle size reduction but rather stayed constant around 65 µm. They also suggested that the particle size reduction is more likely a disruption of agglomerated particles than a destruction of single particles. Therefore, it is conceivable that the disruption of particles caused by sonication is limited to a certain particle size. For oat fiber, the particle size stayed almost constant after ultrasound treatment. It is assumed that the mean particle diameter of around 35 µm is “too small” to be further decreased in size by sonication. Another reason could be that the initial fiber particles of the oat compound used are not in an agglomerated state and therefore cannot be further comminuted. Both assumptions can be supported by FE-SEM images in a later section. The phenomenon of increasing particle sizes after ultrasound treatment for some of the investigated fiber substances (Figure 3 and Figure 4) can be explained by the swelling of certain substances. A study comparing the influence of ultrasound on unpurified and purified rice bran revealed that purified rice bran resulted in reduced particle sizes after sonication, whereas unpurified rice bran showed opposing behavior and particle size increased [40]. These findings were explained by the swelling of protein and starch residues. This is transferable for the compounds of this study. CF AQ and OF, representing the most refined compounds with TDF-contents of a minimum of 90% (Table 1), particle size reductions or at least mostly unchanged particle sizes were measured. For the other compounds, remaining substances, such as distinct amounts of proteins and carbohydrates, are found (Table 1), possibly explaining the increase in particle size.

All in all, the measurements of static light scattering for fiber particles should be taken with caution due to the underlying simplifications. Nevertheless, it can be concluded that high-intensity ultrasound influences particle sizes. This influence is dependent on sonication intensity and treatment time, the initial size of the fiber particles, as well as the nature and purity of the fiber compound. For the investigated fiber compounds, a particle size reduction was achieved for CF AQ, whereas for CF Cl, AF, and PF, an increase was observed. Particle sizes of oat fiber were unaffected.

To investigate the effect of ultrasonication on the techno-functional properties of dietary fiber, the water binding capacity (WBC) and swelling capacity (SC) of the different fiber samples were examined. Both capacities represent important properties of dietary fiber because they represent key functionalities in food products as well as in human physiology. Water binding capacity refers to the amount of water retained by fibers under specific conditions. The binding of water can occur via hydrophilic groups of fiber or entrapment in porous areas within the molecular structure [44]. In a previous study by Kalla-Bertholdt et al. [19], all used fiber compounds were already investigated for WBC, except CF Cl. Therefore, they will only be discussed slightly.

**Figure 5:**
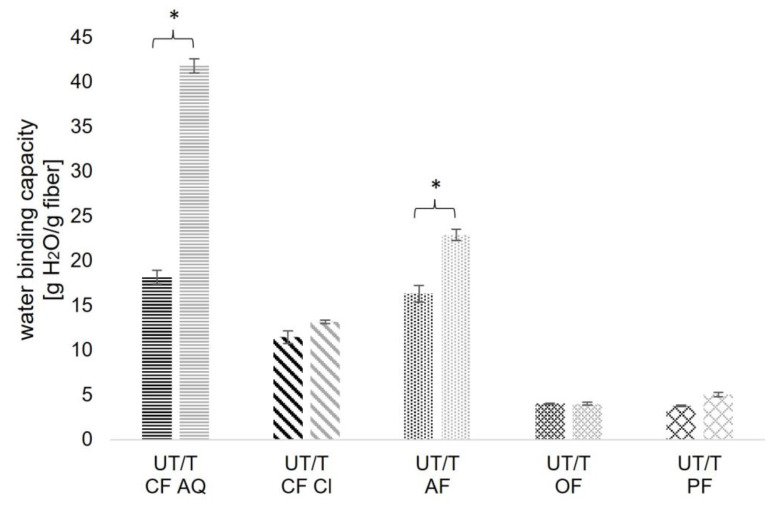
Water binding capacity (WBC) of two different citrus fibers (CF AQ and CF Cl), apple fiber (AF), oat fiber (OF), and pea fiber (PF) before (UT—black) and after ultrasound treatment (T—grey). Columns marked with “*” indicate significant differences between UT and T samples (*p* < 0.05).

WBC is strongly correlated to the chemical structure of the fiber and the functional groups exposed. Functional groups, such as hydroxyl or carbonyl groups, interact with water through hydrogen bonding or dipole interactions, thereby affecting WBC [45]. Additionally, charged groups, often found in pectin, are known to influence the hydration properties of fiber components as well [46]. Furthermore, other parameters like particle size, porosity, and the origin of the fiber compound are additional determining factors [32]. As seen in Figure 5, WBC for all samples showed an increase after US treatment, but only for CF AQ and AF these differences were significant (*p* < 0.05). After sonication, CF AQ more than doubled its WBC (UT: 18.2 g/g; T: 41.84 g/g), and AF showed an increase of around 40% (UT: 16.35 g/g; T: 22.92 g/g). These changes were attributed to cavitation-induced particle size reductions, resulting in a larger surface and therefore more hydrophilic and polar groups accessible for interactions with water [12,19]. For CF Cl, having the largest particle sizes in the raw material (Figure 4), a relatively low WBC was expected, even if the contained soluble dietary fiber content was the highest (Table 2). The slight increase after sonication was not in line with expectations as it was assumed that the remaining pectic substances would bind more water in the matrix after disruption of the cell wall particles. It is known that insoluble dietary fibers, such as cellulose or hemicellulose, are not soluble in water but can retain water in their porous structure or due to interactions with accessible hydrophilic groups. Instead, soluble dietary fibers are soluble in water and are categorized as viscous and non-viscous fibers. Viscous fibers, such as pectins, form a viscous gel after dissolution and therefore increase the water content in the fiber matrix [46]. As visible in the FE-SEM images in a later section, a spider-web-like structure was observed for both untreated and ultrasound-treated samples of CF Cl. Compared to the more open structures of CF AQ and AF, it is assumed that water entrapment in the spider-web-like structures of CF Cl is more difficult than in a layer-like network, as observed for CF AQ and AF after ultrasound treatment.

Considering these investigations, WBC is not only dependent on fiber source, particle size, and SDF and IDF contents but also on the microstructure of fiber particles. Network formation does not necessarily imply improved interactions with water or an increase in water binding capacity.

**Figure 6:**
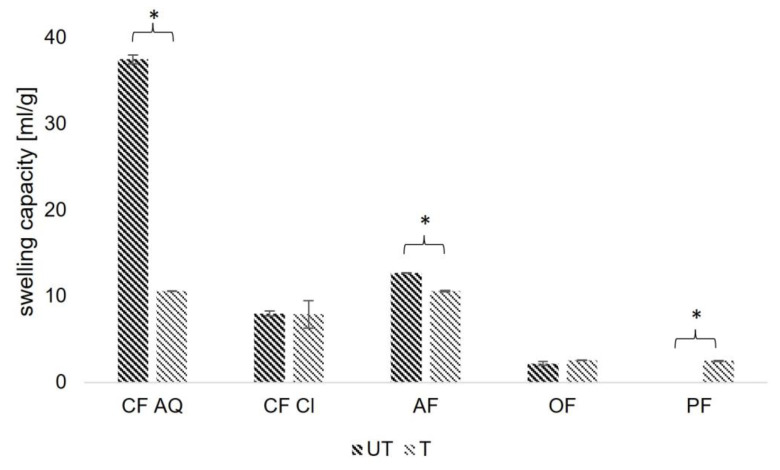
Swelling capacity (SC) of untreated (UT) and treated (T) plant fiber samples (CF AQ—Citrus fiber AQ Plus; CF Cl—Citrus fiber classic; AF—Apple fiber; OF—Oat fiber; PF—Pea fiber). Columns marked with “*” indicate significant differences between UT and T samples (*p* < 0.05).

Swelling capacity measures the variation in the total volume of dietary fiber and water after a distinct period of time. Similar to WBC, this property is closely related to the temperature, origin, chemical structure, particle size, and porosity of the compound [32,47]. In general, there are mostly increasing SCs for mechanically treated dietary fiber compounds found in the literature [23,24,40,48]. For ultrasound treatment, this is attributed to an increase in porosity and the opening of the fiber matrix due to cavitation effects [40]. Water molecules can be bound to exposed hydrophilic groups and incorporated into the fiber matrix, improving its swelling capacity [23]. As seen in Figure 6, the change in SC for untreated and treated fiber components is different. Only for pea fiber is an increase after sonication detectable. This is possibly due to the swelling of remaining starch or protein residues in the compound as well as the disruption of agglomerated particles after sonication, exposing more hydrophilic groups [40,49]. For OF and CF Cl, the SC remained constant, regardless of treatment. As seen in FE-SEM images in a later section, the microstructure, especially the porosity of both samples, was not significantly altered. In a study investigating the effect of high-pressure homogenization on a cellulose-based oat fiber, it was confirmed that SC will not increase with mechanical treatment if structural parameters, such as porosity, are not further modified [50]. This might be transferable, and the underlying reason is that there is no significant effect of ultrasonication on SC for OF and CF Cl. Comparing CF Cl and CF AQ, both having the same origin, it is notable that the SC of purified CF AQ is almost four times as large as that of CF Cl. This phenomenon can be linked to the degree of purification of both fiber compounds. The elimination of impurities, such as starch or protein, is recognized to enhance porosity, resulting in increased surface area and the formation of capillary spaces within the particle matrix. This led to an increase in exposure to more hydroxyl and carboxyl groups, resulting in increased swelling [40]. Regarding the decrease in the SC of CF AQ and AF, no clear underlying connection can be found. Ulbrich & Flöter [50] investigated the effect of high-pressure homogenization and different drying techniques on water binding capacity. It turned out that the effects of mechanical treatment superseded the effects of drying. However, it cannot be excluded that the freeze-drying process had an impact on the results. Additionally, it can be suspected that the milling after lyophilizing destroyed the original microstructure of the ultrasound treatment, having an impact on the results. Further research needs to be conducted.

**Figure 7:**
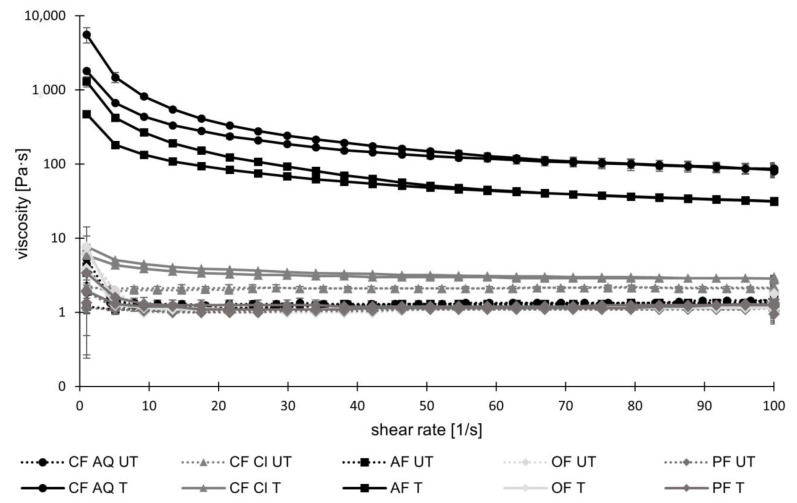
Viscosities of untreated (UT) and treated (T) fiber suspensions, containing 1 wt% dietary fiber suspended in water, as a function of shear rate ranging from 0 to 100 $s^{-1}$ (CF AQ—Citrus fiber AQ Plus; CF Cl—Citrus fiber classic; AF—Apple fiber; OF—Oat fiber; PF—Pea fiber).

To examine viscosity changes after ultrasound treatment, the flow curves of 1 wt% fiber solutions were recorded (Figure 7). In general, the flow curves of all untreated samples showed almost Newtonian fluid behavior. This behavior can be explained by the high amounts of insoluble dietary fiber in all compounds. Cellulose, as the main IDF, is insoluble in water, causing no or minor thickening effects in a solution at low concentrations [51,52]. After ultrasound treatment and with an increasing shear rate, AF and CF AQ exhibited not only non-Newtonian characteristics of a shear-thinning fluid but also increased viscosity. Shear-thinning fluids are characterized by a decreased viscosity with increasing shear rates. Due to entanglement and alignment in the flow direction of tangled molecules, the flow resistance is reduced [53]. While discussing the phenomenon of increased viscosities of CF AQ and AF after US treatment, several studies revealed that sonication of pure pectin solutions leads to decreases in viscosity [54,55,56]. This is explained by the disruption of pectin aggregates, decreasing the chain length of pectin molecules, and therefore resulting in reduced intermolecular interactions [57,58]. As shown in Figure 1, the GalA content, which serves as an indicator for pectic substances, revealed the highest levels in CF Cl, followed by intermediate contents in CF AQ, AF, and PF in descending order, and the lowest amount of GalA in OF. Compared with flow curves demonstrating enormous viscosity increases for CF AQ and AF and nearly constant flow curves for the other three fiber suspensions, it is evident that there is no clear connection between pectic content and viscosity of sonicated samples in this study (Figure 1 and Figure 7). Other studies investigating the rheological properties of strawberry pulp, for example, received similar results of increased viscosity [57]. They suggested that the increase in viscosity is not only attributed to changes in pectin structure but might also be related to the interaction of different substances. Additionally, it was speculated that cell wall material and other contained polysaccharides are solubilized or a better interaction between small particles is achieved due to ultrasound treatment [59,60]. This was also confirmed when evaluating the rheological properties of tomato paste in another study [61]. It was assumed that consistency and particle size are correlated in a complex manner, with larger particles determining higher viscosities while smaller particles reduce viscosity. However, at a critical point, reducing particle size results in higher viscosities due to an increase in total surface area, causing more interactions between particles, or because of the incorporation of smaller particles in the pectin network [61,62]. This could correspond to particle sizes of the raw material used in this study and to particle sizes measured after sonication. CF Cl exhibited the highest amounts of SDF and GalA, indicating the highest pectin content. On the contrary, the particle size was the largest compared to the remaining fiber types. Therefore, it can be assumed that fiber particles were still too large after ultrasound treatment to be incorporated into the pectin network. This could potentially explain the slight increase in viscosity observed for CF Cl. In a recent study by Kalla-Bertholdt et al. [20], the same fiber compound, CF Cl, was sonicated together with pea protein. Following sonication, an increase in viscosity like that of AF was observed. This increase was attributed to the sonication-induced decrease of pea protein particles. These particles serve as fillers within the pectin network, enhancing friction and ultimately causing an increase in flow resistance. However, for CF AQ and AF, particle reduction during sonication appeared to be sufficient in generating a pectin-particle network, resulting in higher viscosities. Additionally, the increase in AIF for CF AQ and AF after sonication (Figure 2) might be another reason for the increase in viscosity (Figure 5), attributed to the gelling of the generated SDF compounds.

All in all, these findings clarify that the determining factors for increased viscosities are not solely the amounts of included viscous components, such as pectin. Instead, particle sizes and particle-particle interactions also play significant roles.

**Figure 8:**
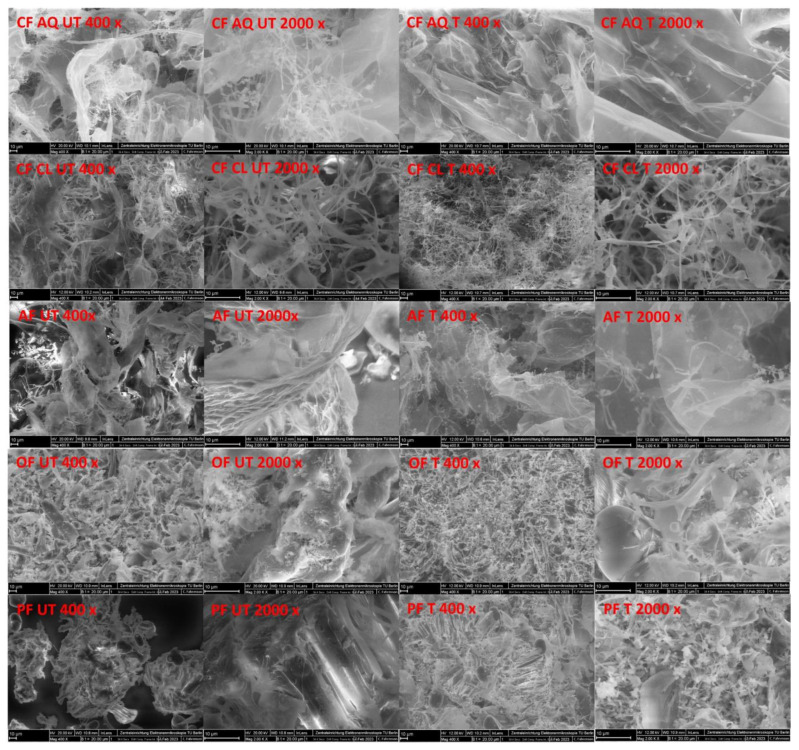
FE-SEM images (400× and 2000× magnification, scale bar 10 µm) of untreated (UT) and ultrasound-treated (T) fiber samples. (CF AQ—Citrus fiber AQ plus, CF Cl—Citrus fiber classic; AF—Apple fiber; OF—Oat fiber, PF—Pea fiber).

The microstructure of the untreated and ultrasound-treated fiber samples is shown in Figure 8. In general, the microstructure of every fiber sample looks different in an untreated state. For PF, typical intact pea hull fragments and remaining starch granules are found [63]. Starch granules are also identified in OF, as well as elongated fiber fragments. For AF, twisted tube-like fiber particles are found, appearing to be very thick and solid. Characteristic thread-like network structures are observed in both CF AQ and CF Cl. For CF AQ, too, very thin sheets are noticeable. After ultrasound treatment, it is obvious that mainly smaller fragments and partly irregular structures occurred. For PF and OF starch granules are remaining, indicating that ultrasound is not affecting its structure. These findings are in accordance with findings in the literature, where ultrasound can create a rough surface of starch granules. Nevertheless, this impact is dependent on the origin of the starch [64,65]. For both OF and PF, the rupture of fiber fragments is the most significant attribute when compared to CF Cl, AF, and CF AQ. Ultrasound generates pressure differences within a solution, resulting in the cavitation of vapour-filled bubbles, which in turn leads to pressure and temperature maxima, microjets, and finally to the disruption of particles [66,67]. For ultrasound treatment of okara DF, it is reported that US treatment changed the irregular, aggregated structure to a decomposed, flaky texture. Due to the destruction of crosslinks between polysaccharide molecules, the cellulose packing of cell wall material is altered, and if the applied energy is high enough, the destruction will take place on the cellular level too [68]. This, in turn, causes a reduction in particle size, as apparent in the FE-SEM images of OF and PF in this study (Figure 8). For CF Cl, the thread-like network structure is still visible after sonication. At 400× magnification, the network seems to be thinner and finer, but at 2000× magnification, no difference is noticed. This indicates that the bigger sheets of the untreated CF Cl sample are thinned during US treatment until only those thread-like structures are left. In a study of ultrasound-treated citrus pectin, similar results were observed. Initially, large and dense blocky structures are partially broken into fragmented branches with loose and thread-like network structures. With increasing frequency, the threads become looser and disrupted due to the increasing cavitation effects, leading to the breakdown of glycosidic bonds [15]. As already stated above, with the disruption of pectin aggregates, the intermolecular interactions are reduced, and functional properties like water binding or gelling, resulting in higher viscous suspensions, are lost [57]. Therefore, it can be assumed that the predominantly thread-like structures in the CF Cl sample are not able to bind or hold water, and no increase in viscosity is achieved (Figure 7). While the untreated CF AQ and AF samples were entirely different, similar structures were found after ultrasound treatment. Both thread-like structures and very thin, nearly transparent layers are observed. In a study investigating the cellulose nanofibers of banana peel after ultrasound treatment, similar microstructures of thin layers were found [17]. It is stated that plant fibers are complex, multilayered structures linked via interfibrillar hydrogen bonds and consisting of aggregated nanofibers. It is possible that, due to sonication-induced cavitation effects, these multilayered aggregated nanofibers are disrupted and individual layers are generated. As already suggested by Chen et al. [57], the increase in viscosity must be related to the interaction of different substances, or in this case, due to the presence of structural components. The thin layers are assumed to be individual cellulose layers, whereas the thread-like structures are suspected to be pectin molecules. The combination of these two structural components may result in a huge ability to bind or incorporate water. Cellulose layers present a larger surface, providing many functional groups for interacting with water. Pectin also presents functional groups, but more importantly, it acts like a net, stabilizing and incorporating water molecules. This results in a swollen matrix, ultimately leading to an increase in viscosity. In a study by Chen et al. [48], DF obtained from grapefruit or orange after enzymatic-ultrasound-assisted extraction were compared. Looser and more complex three-dimensional structures were found in grapefruit, resulting in an increased water-holding capacity. Another reason for the different behavior of AF and CF AQ compared to CF Cl could be found in the production process. All of them are residues of pectin extraction, and as is known, the production process of pectin determines its composition [25]. The first step in pectin extraction is washing the peels of citrus fruits and apples to reduce sugar content and avoid caramelization during processing. Afterwards, an acidic extraction process at pH 1–3 for 3–12 h at 70–100° is conducted. At this stage, the polymerization degree of pectin is reduced, and ester linkages are hydrolyzed, reducing the degree of methylation and acetylation. By tuning t, T, and pH and by using different acids as extraction agents, tailored pectin is formed with the desired properties. In addition to altering the pectin structure during acidic hydrolysis, other cell wall polymers are also affected. Finally, a filtration step is followed to separate the remaining peels and soluble pectin in an aqueous solution [69]. Keeping different extraction methods and their influence not only on pectic substances but also on other cell wall components in mind, it can be assumed that this will greatly impact the functionality of the resulting fiber compounds. It is possible that the insoluble DF, mainly cellulose or hemicellulose, in AF and CF AQ were altered in a way that loosened the multilayered structure. This might have made them more susceptible to being disrupted into individual layers after sonication. For CF Cl the extraction conditions might not have been that intensive because of the more unrefined nature of the compound. Therefore, it can be suggested that the insoluble DF components are present in a different state, reacting differently on sonication.

## 4. Conclusions

The study of the ultrasonication-induced functional and structural modifications in plant fiber has enhanced our understanding of the underlying mechanisms. Ultrasonication, mediated by cavitation effects, influenced key structural attributes such as particle size and the content of alcohol-insoluble fractions, which are an approximation for SDF. The ultrasound treatment caused the disintegration of fiber particles up to a certain threshold, beyond which no further fragmentation was observed. This suggests that ultrasonication predominantly disaggregates particles and does not cleave covalent bonds. However, this general hypothesis may not fully apply to highly purified and pectin-rich fiber compounds, specifically CF AQ and AF. Due to the harsh processing and extraction conditions employed during production, the fiber structure of these compounds may exhibit increased susceptibility to cavitation-induced shear forces, resulting in a transition from IDF to SDF. The SDF content exhibited a notable increase, rising from approximately 5.5% to 17% for CF AQ and from 8% to 17.6% for AF. The transformation of the fiber structure was visually confirmed through FE-SEM images. In the case of CF AQ and AF, the fiber structure evolved from the tube- or thread-like fragments into thin, nearly transparent layered sheets with attached single filaments, possibly indicating the defibrillation of cellulose macrofibrils into microfibrils. This structural alteration had a profound impact on the techno-functional properties, particularly viscosity, which was attributed to the increased SDF content and unique microstructure. Viscosity increased significantly, soaring from approximately 1.4 Pa·s to 84.4 Pa·s for CF AQ and from 1.34 Pa·s to 31.7 Pa·s for AF. In summary, the techno-functional properties of sonicated DF compounds are not solely determined by particle sizes, SDF, or pectin content. Greater emphasis should be placed on the origin of dietary fiber and, most importantly, its processing history. When these factors are considered, ultrasound as a green technology emerges as a promising method for tailoring the functional properties of commercial DF, presenting an interesting approach for the food industry, particularly in the context of clean-labeling purposes.

## Figures and Tables

**Table 1:** Fat-, carbohydrate-, protein-, total dietary fiber- and salt content for the used plant dietary fiber compounds (CF AQ—Citrus Fiber AQ Plus; CF Cl—Citrus fiber classic; AF—Apple fiber; OF—Oat fiber; PF—Pea fiber).

	Fat Content [%]	Carbohydrate Content [%]	Protein Content [%]	Salt Content [%]	Total Dietary Fiber Content [%]
CF AQ	≤1	≤1	5	1.30	90
CF Cl	<1	6	6	0.40	64–82
AF	≤1	≤1	9	1.00	85
OF	≤1	≤1	≤1	0.10	≥90
PF	1	7	7	0.03	70–90

**Table 2:** Insoluble and soluble dietary fiber content [% DM] of the used plant dietary fiber compounds (CF AQ—Citrus fiber AQ Plus; CF Cl—Citrus fiber classic; AF—Apple fiber; OF—Oat fiber; PF—Pea fiber).

	Insoluble Dietary Fiber Content [% DM]	Soluble Dietary Fiber Content [% DM]
CF AQ	74.56 ± 0.34	14.57 ± 0.83
CF Cl	39.80 ± 0.16	23.30 ± 0.22
AF	71.91 ± 1.05	11.14 ± 0.66
OF	94.27 ± 0.40	0.21 ± 0.50
PF	72.91 ± 0.01	4.39 ± 0.35

**Table 3:** Amount of fiber added to suspensions containing fiber and water in a total mass of 200 g.

Sample	Mass of Fiber in Untreated Suspensions [g/100 g]	Mass of Fiber in Treated Suspensions [g/100 g]
CF AQ	1.00 ± 0.01	0.25 ± 0.01
CF Cl	0.50 ± 0.01	0.50 ± 0.01
AF	1.00 ± 0.01	0.50 ± 0.01
OF	1.00 ± 0.01	1.00 ± 0.01
PF	1.00 ± 0.01	1.00 ± 0.01

## Data Availability

The datasets present in this study are available on request from the corresponding author.
